# The ISGyP Endocervical Adenocarcinoma Project: Master Plan Summary, Acknowledgment of Participants, and Participant Responses to Final Recommendations of the Expert Panels

**DOI:** 10.1097/PGP.0000000000000760

**Published:** 2021-02-09

**Authors:** Naveena Singh, Joseph T. Rabban

**Affiliations:** Department of Cellular Pathology, Barts Health NHS Trust, London (N.S.); and School of Medicine, University of California San Francisco, San Francisco, CA (J.T.R.)

**Keywords:** International Society of Gynecological Pathologists, Endocervical adenocarcinoma, Recommendations

## Abstract

The International Society of Gynecological Pathologists carried out a multifaceted project with the broad aim of improving the pathological reporting of endocervical adenocarcinoma (EAC). The intentions were to promote and align practices with the WHO 2020 classification, which endorses HPV status-based classification of EAC and the Silva pattern-based assessment of HPV-associated EAC, to promote uniformity in applying the recent FIGO staging revisions on cervical carcinoma, and to provide best practice guidelines on all aspects of EAC pathology reporting. To facilitate the use of the new WHO/IECC classification and the Silva system, two online educational portals were set up with training and test sets of scanned slides; these remain available to society members on the ISGyP educational website. In addition, a large international collaborative individual data collection project is ongoing, aiming to ascertain the prognostic value of EAC categories, and to provide a database with the potential to address unanswered questions. A single on-site meeting was held on February 29, 2020 in Los Angeles, in advance of the USCAP Annual Meeting; all other correspondence was by email and through electronic surveys. Project participants were invited to vote and comment on the recommendations contained within the practice guideline articles. The project received an enthusiastic response from pathologists across the world. This report includes an overview and summary of all aspects of the project, a list of participants and the results of polling on practice recommendations.

## BACKGROUND

In 2019, under the leadership of its President, Dr Esther Oliva, the International Society of Gynecological Pathologists (ISGyP) embarked on a multifaceted project on endocervical adenocarcinoma (EAC), in part to synchronize with recent updates introduced by the revised FIGO 2018 staging for cervical carcinoma, the fifth edition of the World Health Organization classification of tumors of the female genital tract and the International Collaboration on Cancer Reporting for cervical cancers. In order to develop, promote and facilitate a uniform and standardized approach to pathologic reporting, the ISGyP EAC project engaged the ISGyP members and convened an expert panel to survey current practices to identify areas of discordance, construct an online training portal using scanned slides, develop best practice guidelines with recommendations for pathology reporting, assess ISGyP members response to the final recommendations via polling, and conduct an international multicenter outcome study. Herein we document the mechanics and timeline of these various project components and acknowledge the ISGyP members who contributed time, effort, and creative input to one or more of them.

## PROJECT OVERVIEW

### Project Aims

The overarching aim of the project was to improve the pathologic reporting of EAC. The specific goals are:

- To understand the spectrum of current global practices in the pathologic evaluation (gross specimen management, diagnosis, classification) and reporting (invasiveness, staging, margins) of endocervical cancer.
- To improve the global reproducibility of EAC classification and pathological reporting by:
  - Developing online self-education modules for applying the new International Endocervical Adenocarcinoma Criteria and Classification (IECC) system and Silva classification system of tumor invasiveness.
  - Developing evidence-based best practice guidelines for gross specimen management, tumor classification, staging and reporting, as well as managing problematic and controversial issues.
- To assess the prognostic significance of WHO/IECC EAC classification and Silva pattern-based classification of HPV-associated adenocarcinomas by conducting an international outcome study.

### Project Components

**FIG. 1:**
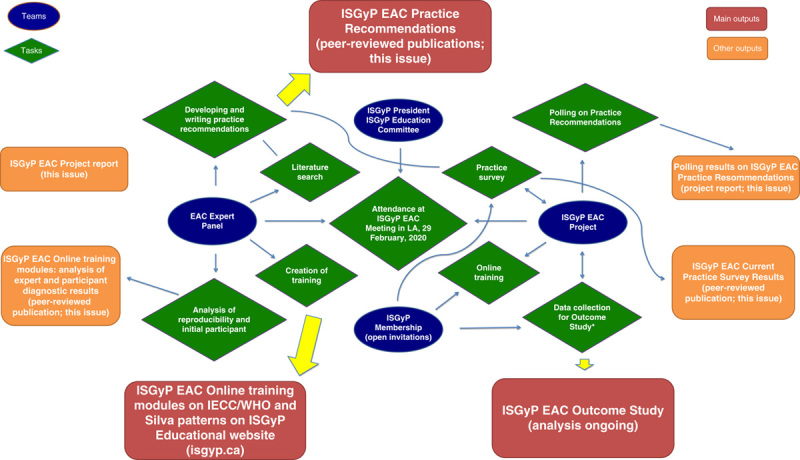
Overview of ISGyP Endocervical Adenocarcinoma (EAC) Project, 2019–2020. IECC indicates International Endocervical Adenocarcinoma Criteria and Classification; ISGyP, International Society of Gynecological Pathologists; WHO, World Health Organization.

An overview of the project components is illustrated in Figure 1.

1. Development of expert panel recommendations.
  A systematic approach to developing the recommendations was implemented, incorporating efforts to identify areas of problems and controversy in real-world practice and to assess the available peer-reviewed literature. This approach engaged the ISGyP membership and was guided by an expert panel in the following sequence:
    (1a) ISGyP member survey on current practices: A survey on current practices for gross specimen management, tumor classification, staging and reporting of EAC was conducted amongst members of ISGyP in 2019 to identify areas of concordance versus discordance. The results are published separately in this issue. The results were presented by the expert panel at a workshop open to ISGyP members in February 2020 in Los Angeles, CA and were used to guide the expert panel’s approach to developing the final guidelines.
    (1b) ISGyP evidence-based recommendations for the pathologic reporting of EAC: An expert panel was established by the ISGyP President to develop evidence-based recommendations for gross specimen management, tumor classification, staging and reporting of EAC. The expert panel was guided in part by input from the ISGyP member survey to identify areas of controversy in real-world practice. The expert panel then identified areas for which a body of peer-reviewed evidence existed and was consistent, areas for which the peer-reviewed evidence was controversial, and areas for which peer-reviewed evidence was lacking. The initial draft of the expert panel recommendations was presented at the workshop open to ISGyP members in February 2020 and open discussion was invited for areas of controversy and areas lacking evidence. Input from these discussions and subsequent deliberations within the expert panel guided the final version of the recommendations, which are published in this issue.
    (1c) ISGyP member response to the expert panel final recommendations: In order to formally acknowledge areas of controversy that persist despite expert panel attempts to achieve consensus, ISGyP members were invited to review the final recommendations and indicate their support or dissent. This was accomplished via online polling. The response rate was 36% among the 245 pathologists involved in the EAC project. The results of the polling did not influence the final recommendations. The poll results are reported in Tables 1–6. The results may be useful for guiding further areas of research.
2. Development of online self-education training modules for applying new tumor classification systems.
  In order to facilitate real-world reproducible application of the new IECC system and Silva system for tumor invasiveness, 2 online training modules were created for self-education by ISGyP members. The modules are hosted on the ISGyP Education Website (www.isgyp.ca) and were made available to ISGyP members in August 2020. Each module contains a description of the diagnostic criteria accompanied by digitally scanned slides organized into a training set of example cases and a separate test set of cases for which the user can submit their interpretation and receive immediate feedback. The reproducibility of the training and test sets among an expert group was evaluated before making the sets available to ISGyP members in August 2020. The results of the reproducibility of the expert group and diagnostic results of participants up to August 2020 are published separately in this issue.
  The modules are available at these sites:
  IECC system for tumor classification module: http://www.gpec.ubc.ca/eac2.
  Silva system for invasiveness module: http://www.gpec.ubc.ca/eac.
3. Development of an international outcome study to evaluate the IECC system and Silva system: In October 2019, the ISGyP Education Committee invited members of ISGyP and the British Association of Gynaecological Pathologists to participate in a collaborative study titled Risk Prediction in Endocervical Adenocarcinoma: International Society of Gynecological Pathologists (ISGyP) Multi Centre Retrospective Observational Study.

**TABLE 1:** Polling results on WHO/International Endocervical Adenocarcinoma Criteria and Classification (IECC) EAC classification recommendations (88 responses)

	Response (%)			
Recommendation	Agree, core	Agree, noncore	Disagree	Other
1. Endocervical adenocarcinomas should be classified according to the forthcoming WHO 2020 classification system, which incorporates the IECC system	94	6	0	0
2. Both classification systems categorize endocervical adenocarcinomas into HPV-associated and HPV-independent types using morphology alone	69	13	9	9
3. Not all HPV-associated endocervical adenocarcinomas, however, have easily identifiable mitoses and apoptotic bodies; in difficult cases, additional tests may be necessary	68	26	0	6
4. The WHO 2020 and IECC systems incorporate newly described microscopic variants of HPV-associated endocervical adenocarcinomas	67	30	1	2
5. Both HPV-associated and HPV-independent endocervical adenocarcinomas can be further stratified using existing criteria. Ancillary testing for diagnosis (such as p16) does not need be reflexively performed on all tumors since morphology is tightly linked to HPV status. p16 and high-risk HPV testing should be reserved for difficult or ambiguous cases	66	24	5	5
6. If interpretation is difficult, a diagnostic algorithm based on the amount of intracytoplasmic mucin and ancillary analyses (such as HPV testing, p16, and GATA3 immunohistochemistry) may be useful in the differential diagnosis of the various histologic types	60	38	0	2
7. RNA-based in situ hybridization for high-risk HPV exhibits higher sensitivity and specificity compared with HPV DNA polymerase chain reaction (PCR)	26	50	16	8
8. RNA-based in situ hybridization (although not available in most institutions) for high-risk HPV has superior sensitivity, specificity, and positive and negative predictive values compared with p16 in identifying HPV-associated endocervical adenocarcinomas	28	52	14	6
9. HPV-associated endocervical adenocarcinomas: usual-type tumors lacking intracytoplasmic mucin should not be diagnosed as endometrioid-type tumors	82	15	2	1
10. HPV-associated endocervical adenocarcinomas: HPV-associated endocervical adenocarcinomas with villoglandular and micropapillary patterns can be designated as usual-type tumors, but these patterns should be noted on the pathology report	51	45	1	3
11. HPV-associated endocervical adenocarcinomas: a diagnosis of primary cervical serous carcinoma should not be made; when dealing with serous-like morphology, most examples will represent an HPV-associated endocervical adenocarcinoma with serous-like morphology or a metastasis from the uterine corpus or fallopian tube/ovary	63	30	3	4
12. HPV-associated endocervical adenocarcinomas: a micropapillary component of any percentage has a propensity for aggressive behavior and should be reported	56	38	2	4
13. HPV-associated endocervical adenocarcinomas: invasive stratified mucinous carcinoma of any percentage has a propensity for aggressive behavior and should be reported	48	42	6	4
14. HPV-associated endocervical adenocarcinomas: mucinous-type (including NOS) tumors are likely associated with a worse survival compared with usual-type tumors, so keeping these 2 categories distinct and reporting them separately is recommended until more studies are conducted	42	44	8	6
15. HPV-associated endocervical adenocarcinomas: while p16 and HPV testing are not always needed, they may be useful in problematic cases, and in the absence of block-type p16 staining or HPV, a diagnosis of HPV-associated endocervical adenocarcinoma should be questioned	67	26	3	4
16. HPV-independent endocervical adenocarcinomas: immunohistochemistry is of limited value in distinguishing between LEGH and well-differentiated variants of gastric-type endocervical adenocarcinoma, and this is a predominantly morphologic diagnosis	57	39	3	1
17. HPV-independent endocervical adenocarcinomas: serous carcinoma of the cervix does not exist	50	25	15	10
18. HPV-independent endocervical adenocarcinomas: true endometrioid adenocarcinoma of the endocervix is extremely rare and should not be reflexively diagnosed in the presence of a mucin-poor endocervical adenocarcinoma	73	24	0	3
19. HPV-independent endocervical adenocarcinomas: immunohistochemical markers are useful for differentiating between the various HPV-independent histologic types, and HPV testing for differentiating between HPV-associated and HPV-independent endocervical adenocarcinomas	59	32	8	1

HPV indicates human papillomavirus; LEGH, lobular endocervical glandular hyperplasia; NOS, not otherwise specified.

**TABLE 2:** Polling results on Silva pattern-based classification of HPV-associated EAC recommendations (83 responses)

	Response (%)			
Recommendation	Agree, core	Agree, noncore	Disagree	Other
1. The pattern-cased classification should be applied to all invasive HPV-associated EAC. The pattern should be included in the final diagnosis and/or comment sections of the surgical pathology report	71	22	1	6
2. If pattern C is identified, the presence of the micropapillary subtype should be reported	54	41	2	3
3. To increase reproducibility, completion of training modules (such as the ISGyP online module on pattern-based classification) and intradepartmental/interdepartmental consultation with colleagues is encouraged	49	45	4	2
4. On excisional specimens, application of the Silva system requires exhaustive microscopic examination of the tumor	43	29	15	13
5. On excisional material, a pattern A designation requires first examination of the entire tumor (to exclude destructive invasion)	82	10	4	4
6. In biopsy material: if present, pattern C can be reported. Pattern A or B designation is not recommended	61	17	10	12
7. As it influences management, it is recommended to report the LVI status in all patients with pattern B and C endocervical adenocarcinoma	96	0	1	3
8. A quantitative estimation of LVI extent can be included in a comment (number of foci)	25	55	13	7
9. Silva pattern-based classification applies only to HPV-associated invasive endocervical adenocarcinomas. Silva pattern-based classification is not recommended in HPV-independent adenocarcinomas	76	17	5	2
10. Invasive adenocarcinoma: in the presence of destructive growth (patterns B and C), diagnosis of invasive adenocarcinoma is warranted	90	3	4	3
11. Invasive adenocarcinoma: in the absence of destructive growth, the following diagnoses should be considered: either adenocarcinoma in situ: in the absence of destructive growth, draw attention to the gland distribution and density; if these are within the confines of the normal endocervix, diagnosis of adenocarcinoma in situ is warranted. Comparison with the uninvolved/normal endocervical gland architecture is helpful. OR pattern-A (nondestructive) adenocarcinoma: if a nondestructive lesion exceeds the size and distribution expected for AIS, or such determination cannot be made, the diagnosis of pattern-A adenocarcinoma (with nondestructive growth) is warranted. It is recommended for now to separate these lesions from frankly invasive adenocarcinoma, as their behavior is largely indolent. It is currently not recommended to classify them as AIS until new evidence on their risk of ovarian spread is available. Reporting size, stage, and margin status is still warranted in this category	84	5	4	7
12. Distinction between in situ and invasive gastric type endocervical adenocarcinoma: in the absence of destructive growth, the following diagnoses should be considered: (i) AIS: gland distribution and density similar and within the confines of the normal endocervix. Comparison with the uninvolved/normal endocervical mucosa is helpful. (ii) Atypical lobular endocervical glandular hyperplasia: floret-like arrangements with small, acini-like glands surrounding a central duct and nuclear atypia. Invasive gastric type adenocarcinoma, minimal deviation type: haphazard distribution of glands with involvement of the deep cervical stroma, lack of lobular organization, minimal to absent nuclear atypia	69	17	4	10

AIS indicates adenocarcinoma in situ; EAC, endocervical adenocarcinomas; HPV, human papillomavirus.

**TABLE 3:** Polling results on grading of EAC recommendations (80 responses)

	Response (%)			
Recommendation	Agree, core	Agree, noncore	Disagree	Other
1. HPV-associated (HPVA) EAC (with some exceptions) should be graded using a combination of architecture and cytology	53	21	19	7
2. HPVA EAC with <10% solid growth are grade 1, 11%–50% solid growth grade 2 and >50% solid growth grade 3. Tumors can be upgraded in the presence of marked nuclear atypia involving >50% of the tumor	41	20	30	9
3. HPV-independent adenocarcinomas should not be graded; in particular, gastric-type adenocarcinomas should not be graded but considered high-grade regardless of morphology	76	9	10	5
4. Endocervical adenocarcinoma admixed with neuroendocrine carcinoma should not be graded but considered high-grade regardless of morphology	83	10	6	1

EAC indicates endocervical adenocarcinomas; HPV, human papillomavirus.

**TABLE 4:** Polling results on predictive biomarkers in EAC recommendations (79 responses)

	Response (%)			
Recommendation	Agree, core	Agree, noncore	Disagree	Other
1. Expert gynecologic pathologists should take the lead in developing robust guidelines for testing and scoring HER2 and PDL1 immunohistochemistry to facilitate standardization in clinical trials. It is strongly recommended to interpret and report predictive biomarkers to response of treatment in endocervical adenocarcinoma in correlation with well-established pathologic parameters	63	28	1	7
2. Until specific recommendations are validated for endocervical adenocarcinoma, prediction of immunotherapy response criteria is identical to that for squamous cervical cancer. At present, PD-L1 immunohistochemistry (CPS of 1 or higher), as determined by the FDA approved companion test, by 22C3 clone, is recommended for pembrolizumab treatment of patients with recurrent or metastatic cervical cancer with disease progression on or after chemotherapy	62	28	0	10
3. With the exception of PD-L1, and based on the lack of scientific evidence at the present time, no other biomarker is recommended for prediction of treatment response in endocervical adenocarcinoma	60	32	3	5
4. Clinical trials specifically designed for HPV-associated and HPV-independent endocervical adenocarcinoma patients are strongly encouraged to elucidate the predictive value of some biomarkers (ERBB2 PD-L1, and others). Trials combining unbalanced number of patients with adenocarcinoma (including HPV-independent disease) and squamous cell carcinoma may yield results not necessarily applicable to endocervical adenocarcinoma patients	62	32	3	3
5. Involvement of expert gynecologic pathologists in the design of future clinical trials is strongly recommended to appropriately identify new predictive biomarkers in cervical adenocarcinoma	80	18	0	2

EAC indicates endocervical adenocarcinomas; HPV, human papillomavirus.

**TABLE 5:** Polling results on EAC staging recommendations (79 responses)

	Response (%)			
Recommendation	Agree, core	Agree, noncore	Disagree	Other
1. Stage should be included in pathology reports as pathologic stage only (pT) based on all available pathologic material	77	10	3	10
2. The staging system used (FIGO 2018, TNM/UICC or both) depends on local practice as some institutions require College of American Pathologists (CAP) AJCC reporting. The staging system used should be indicated alongside the assigned stage	85	10	3	2
3. Use all available material to best assess tumor size, which may require combined gross and microscopic measurements—do not provide separate gross and microscopic dimensions as this causes confusion	84	5	4	7
4. Clinical, pathologic and radiologic assessment can all be used with pathology being the ultimate arbiter of tumor size	71	13	10	6
5. Clinical examination should be used to stage if pathology and radiology are not available	63	25	6	6
6. Depth of invasion should be reported as measured on each specimen but for final staging purposes, the deepest invasion in any single specimen should be used	90	5	1	4
7. Both the largest tumor dimension and depth of stromal infiltration should be provided in the pathology report, with a comment detailing how each measurement was derived	79	13	1	7
8. Total cervical wall thickness in the area of deepest invasion should be reported	61	29	4	6
9. Exophytic tumors should be staged based on the largest tumor dimension, even if superficially invasive (<5 mm)	53	11	23	13
10. Each invasive focus should be measured individually if they are: (i) located in different blocks separated by intervening uninvolved blocks; or (ii) located on separate cervical lips with discontinuous tumor (not involving the curvature of the canal); or (iii) situated at least 2 mm apart in the same section	58	23	11	8
11. At least 2 measurements should be reported: (i) depth of invasion, and (ii) largest tumor dimension	95	1	0	4
12. For circumferential “barrel”-shaped cervix without grossly visible tumor: (i) if tumor is in every quadrant with full thickness invasion of cervical wall, measure the diameter of cervix as closest approximation of tumor size; (ii) for tumors without full thickness invasion and/or tumor in every quadrant, report the deepest invasion, largest tumor dimension as measured histologically and number of quadrants involved with a comment regarding lack of grossly measurable tumor	73	23	3	1
13. Provide entire cervical wall thickness in area of deepest invasion (to calculate % depth of invasion)	58	30	5	7
14. Do NOT use the term “microinvasive carcinoma”	79	11	6	4
15. Measure the tumor as accurately as possible and use the specific FIGO or TNM stage	87	5	3	5
16. For lesions which are a combination of adenocarcinoma in-situ (AIS) and adenocarcinoma: incorporate maximum tumor dimension and assessment of invasive depth as accurately as possible	77	14	3	6
17. For lesions which are a combination of AIS and adenocarcinoma: maximum tumor dimension should form basis of staging	58	11	22	9
18. Tumors should not be staged IB based only on positive margins of a loop or cone excision specimen	72	18	5	5
19. Tumors with positive loop/cone margins should not be staged—in most such cases, there will be a subsequent excision specimen on which the stage can be determined	42	29	14	5
20. Tumor involving the anterior or posterior paracervical tissue, including extension to bladder or bowel WITHOUT mucosal involvement, should be staged as IIB (as this is clinically treated as parametrial involvement)	72	19	4	5
21. Ovarian involvement does not upstage cervical carcinoma, but this should be documented on the pathology report	72	18	1	9
22. Fallopian tube involvement does not upstage cervical carcinoma, but this should be documented on the pathology report; the location of tubal involvement should be documented (mucosal epithelial, mucosal stromal, mural, serosal or intravascular)	71	17	1	11
23. The presence or absence of LVSI should be included in the pathology report	98	2	0	0
24. LVSI at any location, including that seen outside the uterus (eg, parametrial, adnexal) does not upstage a tumor	77	15	1	7
25. The presence of isolated tumor cells (ITCs) in lymph nodes should be recorded but this does NOT count as nodal involvement and should not result in tumor upstaging	68	19	5	8
26. The number of lymph nodes harboring ITCs/micro/macrometastases should be recorded	90	8	1	1
27. When ITCs are scattered throughout the lymph node, measure only contiguous tumor cells to designate ITCs and not aggregate all scattered clusters into a single measurement; however, if multiple collections of ITCs are present, this should be documented in the pathology report	65	23	5	7
28. When there is lymph node involvement, the presence or absence of extracapsular spread should be documented	80	19	0	1

**TABLE 6:** Polling results on EAC grossing recommendations (77 responses)

	Response (%)			
Recommendation	Agree, core	Agree, noncore	Disagree	Other
1. Fragmented LEEP/LLETZ specimens: The presence of thermal artifact on microscopic examination of the tissue edges of a fragmented LEEP specimen is the best marker of a true surgical margin	48	30	12	10
2. Fragmented LEEP/LLETZ specimens: Document the number of tissue fragments and size range (minimum to maximum)	62	27	5	6
3. Fragmented LEEP/LLETZ specimens: Slice the tissue in 2–3 mm thick slices parallel to the endocervical canal	78	16	4	2
4. Fragmented LEEP/LLETZ specimens: Limit the number of slices per cassette in order to avoid incomplete representation due to sectioning artifacts	61	23	9	7
5. Fragmented LEEP/LLETZ specimens: A single H&E-stained section per block is sufficient for initial microscopic examination, with consideration for deeper sections when there is missing mucosa, absence of squamous intraepithelial lesion/endocervical adenocarcinoma, suspicion for stromal invasion, or findings that are discordant with the clinical, colposcopic and/or cytologic findings. Alternatively it may be more efficient to routinely examine 2 or more sections per block, depending on the local practice	57	22	3	18
6. Intact LEEP and cold knife cone specimens: Ink the ectocervical and endocervical mucosal margins as well as the deep connective tissue margin	71	21	4	4
7. Intact LEEP and cold knife cone specimens: If anatomic orientation of the specimen is designated, preserve this orientation in the tissue block designations	75	21	1	3
8. Intact LEEP and cold knife cone specimens: Document length, diameter and wall thickness of the specimen	77	17	1	5
9. Intact LEEP and cold knife cone specimens: Document the anatomic location of tumor in the cervix using positions on a clock or designating anterior versus posterior lip of the cervix	73	20	5	2
10. Intact LEEP and cold knife cone specimens: Document the distance of tumor to the endocervical margin, ectocervical margin, and deep connective tissue margin	74	17	4	5
11. Intact LEEP and cold knife cone specimens: Document the tumor length (parallel to the endocervical canal), tumor width (perpendicular to the endocervical canal), tumor thickness and depth of tumor invasion	74	12	5	9
12. Intact LEEP and cold knife cone specimens: Fresh intact LEEP/cone specimens can either be opened and pinned before fixation or placed intact in formalin, depending on local practice resources	47	38	8	7
13. Intact LEEP and cold knife cone specimens: Specimens opened and pinned before fixation should be thinly sliced parallel to the endocervical canal	55	27	13	5
14. Intact LEEP and cold knife cone specimens: Specimens fixed intact can be sliced using a radial or a parallel slicing strategy	55	29	9	7
15. Intact LEEP and cold knife cone specimens: Specimens should be enterly submitted for microscopic examination, including any excess trimmed pieces	82	9	2	7
16. Intact LEEP and cold knife cone specimens: If an additional so-called top hat specimen is submitted, it should be inked, sliced using the same strategy for the main LEEP specimen, and entirely submitted	75	16	1	8
17. Intact LEEP and cold knife cone specimens: A single H&E-stained section per block is sufficient for initial microscopic examination, with consideration of deeper sections when there is missing mucosa, absence of squamous intraepithelial lesion/endocervical adenocarcinoma, suspicion for stromal invasion, or findings that are discordant with the clinical, colposcopic and/or cytologic findings. Alternatively it may be more efficient to routinely examine 2 or more sections per block, depending on the local practice	58	21	5	16
18. Trachelectomy specimens: Orient the laterality of the parametria and the anterior/posterior lip of the cervix based on the orientation provided by the surgeon	78	17	0	5
19. Trachelectomy specimens: Ink the endocervical, vaginal, and parametrial margins, as well as the nonperitonealized connective tissue at the outer surface of the anterior and posterior cervical walls	81	10	3	6
20. Trachelectomy specimens: Measure the cervix length (parallel to the endocervical canal), diameter and wall thickness	78	16	0	6
21. Trachelectomy specimens: Measure the parametrial tissue length (from superior to inferior) and lateral dimension (from uterine wall to distal edge)	66	25	1	8
22. Trachelectomy specimens: Measure the vaginal cuff minimal and maximal length after stretching it out if it is retracted	74	17	1	8
23. Trachelectomy specimens: Document the anatomic location of tumor in the cervix using positions on a clock face and then correlate with the anatomic terminology used by the surgeon	73	17	4	6
24. Trachelectomy specimens: Document the distance of tumor to the endocervical margin, vaginal margin, parametrial margin, and nonperitonealized connective tissue at the outer surface of the anterior and posterior cervix walls	73	16	3	8
25. Trachelectomy specimens: Document the tumor length (parallel to the endocervical canal), tumor width (in the plane perpendicular to the endocervical canal), tumor thickness and depth of tumor invasion	75	8	4	13
26. Trachelectomy specimens: Remove parametria and place in cassettes before opening specimen	42	27	17	14
27. Trachelectomy specimens: Open the specimen and obtain measurements (anatomic structures and any lesion) immediately upon receipt	35	33	20	12
28. Trachelectomy specimens: After intraoperative consultation (if performed), pin the specimen for overnight formalin fixation, taking care to stretch out the vaginal cuff to its full length and pin	47	30	12	11
29. Trachelectomy specimens: Tumors 2 cm or less should be entirely submitted while tumors larger than 2 cm can be processed using representative sections	57	26	3	14
30. Trachelectomy specimens: Tissue sections should demonstrate the deepest tumor invasion and the closest approach of the tumor to the vaginal, radial, and parametrial margins	85	8	0	7
31. Trachelectomy specimens: If there is no grossly visible lesion, the entire cervix should be submitted	84	7	1	8
32. Trachelectomy specimens: Perpendicular sections of the vaginal margin closest to the tumor should be examined. Whether the remainder of the vaginal margin should be examined entirely en face or by representative perpendicular sections is left to local practice standards. Similarly, if there is no macroscopic tumor, the decision to examine the entire vaginal margin en face or by representative perpendicular sections is left to local practice standards	68	25	0	7
33. Trachelectomy specimens: The parametria should be entirely submitted	69	14	7	10
34. Trachelectomy specimens: A single H&E-stained section per block is sufficient for initial microscopic examination	65	26	1	8
35. Trachelectomy specimens: The protocol for intraoperative evaluation of trachelectomy specimen should be decided at the local practice level using 1 of the 4 published protocols 24–27, in conjunction with discussion with the surgeon regarding their specific intraoperative needs	47	38	7	8
36. Trachelectomy specimens: The presence or absence of invasive cancer and of in situ carcinoma at the proximal margin (defined as ink on tumor) should be reported for the intraoperative evaluation	66	18	5	11
37. Hysterectomy specimens: Ink the vaginal and parametrial margins, as well as the nonperitonealized connective tissue at the outer surface of the anterior and posterior cervical walls	81	14	1	4
38. Hysterectomy specimens: Weigh the uterus	48	29	18	5
39. Hysterectomy specimens: Measure the cervix length (parallel to the endocervical canal), diameter and wall thickness	66	26	7	1
40. Hysterectomy specimens: Measure the parametrial tissue length (from superior to inferior) and lateral dimension (from uterine wall to distal edge)	60	31	7	2
41. Hysterectomy specimens: Measure the vaginal cuff minimal and maximal length after stretching it out if it is retracted	75	20	3	2
42. Hysterectomy specimens: Measure the uterine corpus from superior to inferior, side to side and anterior to posterior dimensions	75	21	3	1
43. Hysterectomy specimens: If present, the size of the ovaries and fallopian tubes should be recorded	78	22	0	0
44. Hysterectomy specimens: If there is no suspicion that the tumor is extending into the parametria, then they can be removed at the interface with the uterine wall, sliced at 2–3 mm intervals and placed in tissue cassettes before opening the uterus. Otherwise the parametria should be left attached and sliced in continuity with the cervix	58	34	4	4
45. Hysterectomy specimens: Open the uterus immediately upon receipt in the lab in order to begin formalin fixation	58	26	7	9
46. Hysterectomy specimens: Fresh hysterectomy specimens can be opened either by amputating the cervix and processing it like a trachelectomy or by the conventional bivalve strategy for opening a uterus	57	22	8	13
47. Hysterectomy specimens: Slice the uterine corpus in parallel thin slices before formalin fixation	23	26	43	8
48. Hysterectomy specimens: Stretch out the vaginal cuff to its full length and pin in position before formalin fixation	41	30	29	0
49. Hysterectomy specimens: Overnight formalin fixation is advised before further tissue sampling	58	26	16	0
50. Hysterectomy specimens: Document tumor involvement in relation to the endocervix, ectocervix, parametria, and uterine corpus	87	12	0	1
51. Hysterectomy specimens: Document the anatomic location of tumor in the cervix using positions on a clock or designating anterior versus posterior lip of the cervix	74	22	3	1
52. Hysterectomy specimens: Document distance of tumor to the vaginal margin, parametrial margin, and nonperitonealized connective tissue at the outer surface of the anterior and posterior cervix walls	82	13	4	1
53. Hysterectomy specimens: Document the tumor length (parallel to the endocervical canal), tumor width (in the plance perpendicular to the endocervical canal), tumor thickness and depth of tumor invasion	81	14	0	5
54. Hysterectomy specimens: If the cervix was amputated, opened, and pinned out before fixation, then make 2–3 mm slices parallel to the endocervical canal	70	17	8	5
55. Hysterectomy specimens: If the cervix was not amputated and pinned out before fixation, then perform radial slices at 2–3 mm intervals parallel to the endocervical canal	62	13	18	7
56. Hysterectomy specimens: Tumors 2 cm or less should be entirely submitted, whereas tumors larger than 2 cm can be representatively sampled	70	21	3	6
57. Hysterectomy specimens: Tissue sections should particularly target tumor in relation to closest vaginal, paracervical/radial, and parametrial margins	85	13	1	1
58. Hysterectomy specimens: If there is no grossly visible lesion, the entire cervix should be submitted	87	11	1	1
59. Hysterectomy specimens: Perpendicular sections of the vaginal margin closest to the tumor should be examined. Whether the remainder of the vaginal margin should be examined entirely en face or by representative perpendicular sections is left to local practice standards. Similarly, if there is no macroscopic tumor, the decision to examine the entire vaginal margin en face or by representative perpendicular sections is left to local practice standards	70	25	1	4
60. Hysterectomy specimens: The parametria should be entirely submitted	70	16	7	7
61. Hysterectomy specimens: The full thickness of the anterior and posterior walls of the corpus and of the lower uterine segment should be representatively sampled	71	22	7	0
62. Hysterectomy specimens: The fallopian tubes should be processed using the SEE-Fim protocol and the fimbriae should be entirely submitted while the ampullary portion can be representatively sampled	44	35	13	9
63. Hysterectomy specimens: The ovaries can be representatively sampled	64	32	0	4
64. Pelvic exenteration specimens: Identify all anatomic structures present (cervix, uterine corpus, vagina, urinary bladder, rectum) in conjunction with the operative note or discussion with the surgeon	82	14	0	4
65. Pelvic exenteration specimens: Margins to ink are the vagina, parametria, urethra, ureters, proximal and distal rectal margins, and soft tissue margins	75	12	4	9
66. Pelvic exenteration specimens: Measure all the organs in the fresh state	31	32	20	17
67. Pelvic exenteration specimens: Inflate the urinary bladder and rectum with formalin for several hours or overnight and then hemisect the specimen	23	30	20	27
68. Pelvic exenteration specimens: Measure the tumor in 3 dimensions and document its relationship to all the organs and margins	79	14	0	7
69. Pelvic exenteration specimens: Representative sections of the tumor should demonstrate its relationship to all organs and margins	83	9	0	8
70. Pelvic exenteration specimens: The vaginal and parametrial margins are processed as is done for a hysterectomy specimen	75	16	3	6
71. Pelvic exenteration specimens: The urethral, ureteral, rectal, and soft tissue margins are processed en face, unless there is tumor nearby in which case perpendicular margins are advised	71	21	1	7
72. Lymph node specimens: Record the size and number of macroscopically detectable lymph nodes	75	13	3	9
73. Lymph node specimens: Remove excess adipose tissue from lymph nodes (nonsentinel and sentinel) and slice perpendicular to long axis at 2 mm intervals	70	20	3	7
74. Lymph node specimens: Submit all slices of each lymph node for microscopic examination unless there is an obvious macroscopic metastasis, in which case representative section is sufficient	73	14	3	10
75. Lymph node specimens: Excess adipose tissue trimmed away does not need to be submitted for microscopic examination	42	35	17	6
76. Lymph node specimens: Document the number of lymph nodes in each cassette to allow for an accurate total count	79	14	1	6
77. Lymph node specimens: If no lymph nodes are identified grossly, submit the entire tissue for microscopic examination	72	18	5	5
78. Lymph node specimens: For nonsentinel nodes, a single H&E-stained section per tissue block is sufficient	73	23	1	3
79. Lymph node specimens: For sentinel nodes, ultrastaging by deeper level sections should be performed; however, the number of levels and the distance between levels should be decided by local practice conditions as there is insufficient evidence to make a specific recommendation	68	27	3	2
80. Lymph node specimens: Intraoperative evaluation of SLN should be performed only if the surgeon is prepared to alter the intraoperative plan based on the results and is aware of the limitations to diagnostic sensitivity	74	24	1	1
81. Lymph node specimens: After removing excess adipose tissue, slice the SLN perpendicular to long axis at 2 mm intervals and evaluate all slices by frozen section	56	20	13	11
82. Lymph node specimens: Do not perform deeper levels intraoperatively except to pursue suspicious findings	52	37	7	4
83. Lymph node specimens: Apply the standard ultrastaging protocol for permanent section processing of the remainder of the frozen tissue block	68	23	5	4

EAC indicates endocervical adenocarcinomas; HPV, human papillomavirus; LEEP/LLETZ, loop electrosurgical excision procedure/large loop excision of the transformation zone.

The study was designed to evaluate patients with EAC for whom a minimum of 2 yr of follow-up is available and to correlate outcome with the IECC system and Silva system for tumor invasiveness. Assessment of the IECC tumor classification and Silva pattern of invasiveness was performed locally by the contributing institution. Each participating institution was requested to contribute a minimum of 15 consecutive cases with complete data. The project received sponsorship from Queen Mary University of London on December 2, 2019, and ethical approval from the South West-Frenchay Research Ethics Committee on February 26, 2020 (IRAS 273971; REC ref 20/S/0008). Data accrual was closed in October, 2020. Due to delays as a result of the pandemic, completion of paperwork, data cleaning and analysis are currently ongoing and preliminary results are expected in 2021.

### Acknowledgement of Project Participants

The success of the ISGyP EAC Project is a result of more than 250 pathologists across the globe who have dedicated time, effort, and creative input to various components of the project. These pathologists are acknowledged in Table 7. Gratitude is also extended to several pathologists who preferred to remain anonymous. All contributors from each Centre participating in the outcome study will be acknowledged in the publication(s) arising from the data; only the pathologists from these Centres are listed in Table 7.

**TABLE 7:** List of International Society of Gynecological Pathologists (ISGyP) endocervical adenocarcinoma (EAC) expert panel and project participants*†, and ISGyP education committee

ISGyP EAC expert panel		
Abu-Rustum	Nadeem	USA
Alvarado-Cabrero	Isabel	Mexico
Bosse	Tjalling	The Netherlands
Ellenson	Lora H	USA
Gilks	C Blake	Canada
Kiyokawa	Takako	Japan
Lax	Sigurd	Austria
Malpica	Anais	USA
Matias-Guiu	Xavier	Spain
McCluggage	W Glenn	UK
Oliva‡	Esther	USA
Park	Kay J	USA
Parra-Herran	Carlos	Canada
Rabban	Joseph T	USA
Ramirez	Pedro T	USA
Roma	Andres	USA
Singh	Naveena	UK
Soslow	Robert	USA
Stolnicu	Simona	Romania
Talia	Karen	Australia
Zannoni	Gian Franco	Italy
ISGyP EAC Project Participants*†		
Abu-Sinn	Dua	UK
Adler	Esther	USA
Abu-Rustum	Nadeem	USA
Akakpo	Kafui	Ghana
Ali-Fehmi	Rouba	USA
Alvarado-Cabrero	Isabel	Mexico
Amaker	Barbara	USA
Anderson	Lyndal	Australia
Arafah	Maria	Saudi Arabia
Ardighieri	Laura	Italy
Arif	Saimah	UK
Arora	Rupali	UK
Arseneau	Jocelyne	Canada
Atez	Denis	Turkey
Attygalle	Ayoma	UK
Balzer	Bonnie	USA
Banet	Natalie	USA
Barroeta	Julieta	USA
Bartosch	Carla	Portugal
Basheska	Neli	North Macedonia
Bell	Karen	USA
Bennett	Jennifer	USA
Bergeron	Christine	France
Bhatnagar	Anjali	UK
Bittinger	Sophie	Australia
Bleeker	Maaike	The Netherlands
Bosse	Tjalling	The Netherlands
Brainard	Jennifer	USA
Bryant	Anna	UK
Burandt	Eike-Christian	Germany
Buza	Natalia	USA
Cardinal	Lucia H	Argentina
Carinelli	Silvestro	Italy
Carlson	Joseph	Sweden
Chan	Joanna	USA
Ching	Yeo Yen	Singapore
Clarke	Blaise	Canada
Cohen	Paul	Australia
Colpaert	Cecile	Belgium
Conlon	Niamh	Ireland
Costa	Irmgard	Spain
Coutts	Michael	UK
Croce	Sabrina	France
Crum	Christopher	USA
Culora	Giuseppe	UK
Cummings	Margaret	Australia
Desai	Sudha	UK
Dillon	Jessica	USA
Djordevic	Bojana	Canada
Duggan	Maire	Canada
Dundr	Pavel	Czech Republic
Eaton	Lynn	USA
Ellenson	Lora H	USA
Elliot	Victoria	UK
Ewing	Patricia	The Netherlands
Fadare	Oluwole	USA
Felix	Ana	Portugal
Ferreira	Joana	Portugal
Focchi	Gustavo	Brazil
Ganesan	Raji	UK
Gao	Hongwen	China
Garg	Karuna	USA
Geisinger	Kim	USA
Giannico	Giovanna	USA
Gibbs	Paul	USA
Gilks	C Blake	Canada
Griffin	Brannan	USA
Guarch	Rosa	Spain
Guerra Fernandez	Esther	Spain
Gupta	Mamta	USA
Habanabakize	Thomas	Rwanda
Hadwin	Richard	UK
Hagemann	Ian S	USA
Haider	Shireen	UK
Hardisson	David	Spain
Hasan	Noori	UK
Hoang	Lynn	Canada
Hodgson	Anjelica	Canada
Horn	Lars-Christian	Germany
Hui	Pei	USA
Hyder	Paula	UK
Hyne	Suzanne	Australia
Ibrahim	Samiya	UK
Ip	Philip	Hong Kong
Irving	Julie	Canada
Isacson	Christina	USA
Jacobsen	Anne-Marie	Canada
Jadhav	Nupur	USA
Jarboe	Elke	USA
Jaworski	Richard	Australia
Jaynes	Eleanor	UK
Jiang	Qingping	China
Joehlin-Price	Amy S	USA
Kalinganire	Nadia	Rwanda
Karnezis	Anthony	USA
Kila	Yemisi	Nigeria
Kim	Kyu-Rae	South Korea
Kiyokawa	Takako	Japan
Kolin	David	USA
Kong	Christina	USA
Kooreman	Loes	The Netherlands
Krisztina	Hanley	USA
Lax	Sigurd	Austria
Lebok	Patrick	Germany
Leen	Sarah Lamshang	UK
Lerias	Sofia	Portugal
Liao	Shu Yuan	USA
Lieberman	Richard	USA
Liu	Aijun	China
Llamosas	Fernando	Paraguay
Longacre	Teri A	USA
Lynn	Amy	USA
Mahmoud	Khalifa	USA
Malpica	Anais	USA
Mandalia	Trupti	UK
Manek	Sanjiv	UK
Maniar	Kruti	USA
Masand	Ramya	USA
Masback	Anna	Sweden
Mateoiu	Claudia	Sweden
Matias-Guiu	Xavier	Spain
McCluggage	W Glenn	UK
McDermott	Jacqueline	UK
McGregor	Stephanie	USA
McLachlin	C Meg	Canada
McPhaul	Lauron	USA
Medeiros	Fabiola	USA
Mendoza	Arturo	USA
Merino	Maria J	USA
Mikami	Yoshiki	Japan
Milla	Esperanza	Peru
Mohammed	Umar	Nigeria
Moisini	Ioana	USA
Mom	Stijn	The Netherlands
Mukonoweshuro	Pinias	UK
Murali	Rajmohan	USA
Murphy	Karla	USA
Namasivayam	Jeyalakshmi Devi	India
Newman	Marsali	Australia
Nitch-Smith	Harriet	UK
Nout	Remi	The Netherlands
Oliva	Esther	USA
Ondic	Ondrej	Czech Republic
Ordulu	Zehra	USA
Otis	Christopher	USA
Ozor	Nnamdi Sergius	Nigeria
Paczos	Tamera	USA
Palacios	Jose	Spain
Palicelli	Andrea	Italy
Park	Kay J	USA
Parkash	Vinita	USA
Parra-Herran	Carlos	Canada
Patil	Ninad M	USA
Pesci	Anna	Italy
Phelan	Sine	Ireland
Piazzola	Elena	Italy
Pinto	Andre	USA
Plotkin	Anna	Canada
Policarpio-Nicolas	Maria Luisa	USA
Portillo	Sofia Canete	USA
Post	Miriam	USA
Powell	Aime	Australia
Quddus	Ruhul	USA
Quick	Charles Matthew	USA
Rabban	Joseph T	USA
Rakislova	Natalia	Spain
Ramirez	Pedro T	USA
Rapaz-Lerias	Sofia	Portugal
Rasty	Golnar	Canada
Redline	Raymond	USA
Rekhi	Bharat	India
Richards	Stephanie	USA
Riopel White	Ann	USA
Rivera-Colón	Glorimar	USA
Roma	Andres	USA
Saad	Heba	USA
Saco	Maria Adela	Spain
Safdar	Nida	USA
Sah	Shatrughan	UK
Samra	Spinder	Australia
Schmidt	Dietmar	Germany
Schmitt	Alessandra	USA
Segura	Shiela	USA
Sehdev	Ann	USA
Shukla	Pratibha	USA
Siatecka	Hanna	USA
Silva	Elvio	USA
Singh	Charanjeet	UK
Singh	Kamaljeet	USA
Singh	Naveena	USA
Singh	Neeta	UK
Srinivasan	Radhika	India
Staats	Paul	USA
Staebler	Annette	Germany
Stewart	Colin	Australia
Stolnicu	Simona	Romania
Strickland	Kyle	USA
Sun	Ping-Li	China
Syed	Sheeba	UK
Talia	Karen	Australia
Tan	Adeline	Australia
Tu	Xiaoyu	China
Turashvili	Gulisa	Canada
Usubutun	Alp	Turkey
van de Vijver	Koen	Belgium
van der Griend	Rachael	New Zealand
Van Rompuy	Anne-Sophie	Belgium
Vang	Russell	USA
Varghese	Sharlin	USA
Vella	Jo	UK
Vergine	Marco	UK
Villena	Nadia	Denmark
Volchek	Mila	Australia
Vroobel	Katherine	UK
Wadee	Reubina	South Africa
Watkins	Jaclyn	USA
Webb	Patricia	Peru
Wei	Jian-Jun	USA
Williams	Anthony	UK
Williams	Jonathan	UK
Wise	Olga	UK
Wolsky	Rebecca	USA
Wong	Jason	Hong Kong
Wong	Richard Wing-Cheuk	UK
Wong	Serena	USA
Wong	Tak Siu	Hong Kong
Yemelyanova	Anna	USA
Young	Robert H	USA
Zannoni	Gian Franco	Italy
Zarei	Shabnam	USA
Zhang	Gloria	USA
Zhang	Jing	China
Zheng	Wenxin	USA
Zuna	Rosemary E	USA
ISGyP Education Committee		
Carlson	Joseph	Sweden
Clarke	Blaise	Canada
Fadare	Oluwole	USA
Hui	Pei	USA
Kim	Kyu-Rae	South Korea
Matias-Guiu	Xavier	Spain
Oliva	Esther	USA
Rabban	Joseph	USA
Singh	Naveena	UK

* Includes members of the British Association of Gynaecological Pathologists who came forward to participate in outcome study, and invited participants who are not members of either society.

† Some participants preferred to remain anonymous, or their full names/country of work were unavailable at the time of submission. A complete list of participants who contributed to data collection for the outcome study from each participating Centre will be included in future publication(s).

‡ ISGyP President and Project Lead.

## CONCLUSIONS

The ISGyP EAC Project has now concluded, with the exception of the analysis of the results of the international outcome study. The extensive contributions of pathologists all across the world has enabled the Project to be a success and is a testament to the value of global collaboration. As co-Chairs of the ISGyP Education Committee, the authors take this opportunity to express their gratitude to all participants, whether or not included in the participant list, for making this endeavor a success in the hope that it will contribute to improving the standard of pathology reporting in EAC and thereby improving the clinical outcomes of our patients.

